# *Foxg1* deletion impairs the development of the epithalamus

**DOI:** 10.1186/s13041-018-0350-2

**Published:** 2018-02-02

**Authors:** Bin Liu, Kaixing Zhou, Xiaojing Wu, Chunjie Zhao

**Affiliations:** 1 Key Laboratory of Developmental Genes and Human Diseases, MOE, School of Medicine, Southeast University, Nanjing, 210009 People’s Republic of China; 2 Depression Center, Institute for Brain Disorders, Beijing, 100069 China

**Keywords:** Epithalamus, Habenula, Pineal gland, Choroid plexus, FOXG1-related disorders, Sleep disturbance, *Fgf15*

## Abstract

The epithalamus, which is dorsal to the thalamus, consists of the habenula, pineal gland and third ventricle choroid plexus and plays important roles in the stress response and sleep–wake cycle in vertebrates. During development, the epithalamus arises from the most dorsal part of prosomere 2. However, the mechanism underlying epithalamic development remains largely unknown. *Foxg1* is critical for the development of the telencephalon, but its role in diencephalic development has been under-investigated. Patients suffering from FOXG1-related disorders exhibit severe anxiety, sleep disturbance and choroid plexus cysts, indicating that *Foxg1* likely plays a role in epithalamic development. In this study, we identified the specific expression of *Foxg1* in the developing epithalamus. Using a “self-deletion” approach, we found that the habenula significantly expanded and included an increased number of habenular subtype neurons. The innervations, particularly the habenular commissure, were severely impaired. Meanwhile, the *Foxg1* mutants exhibited a reduced pineal gland and more branched choroid plexus. After ablation of *Foxg1* no obvious changes in Shh and Fgf signalling were observed, suggesting that *Foxg1* regulates the development of the epithalamus without the involvement of Shh and Fgfs. Our findings provide new insights into the regulation of the development of the epithalamus.

## Introduction

The epithalamus, which consists of the habenula, pineal gland, and third ventricle choroid plexus (3^rd^Chp), is involved in many functions, including motor control, the sleep–wake cycle and stress responses [1–3]. The habenula is highly conserved in vertebrates and acts as a critical node connecting the forebrain to the midbrain and hindbrain by receiving inputs from the limbic system and the basal ganglia and projecting to the monoaminergic nuclei [4, 5]. The pineal gland is critical for the regulation of circadian rhythms due to its production of melatonin [6], and the choroid plexus synthesizes cerebrospinal fluid (CSF) and many growth factors, including fibroblasts and insulin-like and platelet-derived growth factors, and plays important roles, such as providing a route for nutrients and removing by-products of metabolism [7, 8]. Dysfunction of the epithalamus has been reported to be related to mood disorders, such as major depression, and schizophrenia and sleeping disorders [2, 9–12]. However, knowledge regarding the developmental process of the epithalamus is limited.

During early development, the progenitor domain in the diencephalon is divided into three prosomeres (p), i.e., p1, p2, and p3, along the anterior-posterior axis [13, 14]. P1 and p3 give rise to the pretectum and prethalamus, respectively. The most dorsal region of p2 produces the epithalamus, and the other part generates the thalamus. In the presumptive epithalamic progenitor domain, the most anterior area containing the roof plate develops into the 3^rd^Chp, while the adjacent part generates the habenular commissure, paired habenulas and pineal gland. Previously, a member of the fibroblast growth factor (Fgf) family, *Fgf8*, has been reported to regulate the development of the habenula and pineal gland in a dose-dependent manner [15]. In zebrafish, Fgf signalling also controls the specification of the pineal complex [16]. However, the molecular and cellular mechanisms underlying the development of the epithalamus still remain largely unknown.

*Foxg1* encodes a winged-helix transcriptional repressor and has been reported to play critical roles during telencephalic development [17–20]. Patients with mutations in *FOXG1* have been reported to suffer from mental retardation, poor social interactions and severe anxiety [21]. Notably, severe sleep disturbance, deformation of the third ventricle and choroid plexus cysts have also been reported [22, 23]. Thus, *Foxg1* may also be involved in the regulation of epithalamic development. In the present study, we found that a disruption of *Foxg1* leads to an impaired epithalamus with an expanded habenula, a smaller pineal gland and an extremely complicated choroid plexus. Various subtypes of neurons in the habenula exhibited a remarkable increase in number with impaired innervations. Furthermore, ablation of *Foxg1* led to the abnormal sub-regionalization of the epithalamic progenitor domain. Our data provide novel perspectives regarding the development of the epithalamus.

## Methods

### Animals

*Foxg1-Cre* (*Foxg1* ^*tm1(cre)Skm*^) [24] mouse line was purchased from the Jackson laboratory (US, *Foxg1-Cre* stock: 006084). The *Foxg1*^*fl/fl*^ line was obtained as previously described [19, 25]. The *Fzd10-EGFP* transgenic line was generated using standard methods (unpublished data). *Foxg1* disruption was achieved by an intercross of *Foxg1-cre* or crossing *Foxg1-cre* with *Foxg1*^*fl/+*^. Both *Foxg1*^*cre-cre*^ *and Foxg1-cre;Foxg1*^*fl/+*^ were considered mutants, and their wild-type littermates and *Foxg1*^*fl/+*^ were considered controls. All animals were maintained on an outbred CD1 genetic background and were housed in the animal facility of the Southeast University. All experimental procedures followed the guidelines approved by Southeast University. To stage the embryos, the mice were mated in the afternoon. The day the vaginal plug was found at noon was considered embryonic day 0.5 (E0.5), and the day of birth was considered postnatal day 0 (P0).

### Tissue processing and Nissl staining

Embryonic brains were directly dissected in cold phosphate buffered saline (PBS) and immediately transferred to 4% paraformaldehyde (PFA, Sigma-Aldrich, 441,244, US) overnight at 4 °C. The brains from P0 were perfused and then post-fixed at 4 °C for 12–16 h. The brains were then cryoprotected in 30% sucrose and embedded in OCT. Coronal sections (8–12 μm thick) were obtained using a Leica cryostat (CM 3050S) and stored at − 70 °C until use. The Nissl staining was performed according to standard protocols.

### In situ hybridization

E12.5 brains were dissected, immediately transferred to 500 μL of TRI Reagent (Sigma-Aldrich, T9424, US) and processed for total RNA isolation according to the manufacturer’s instructions. After purification using the RNeasyPlus Mini Kit (QIAGEN, 74,106, DE), the RNA concentration was measured using an Agilent 2100 Bioanalyser (Agilent Technologies, Palo Alto, CA). In total, 2 μg of purified total RNA was used as the template to synthesize cDNA using the PrimeScript ™ RT Master Mix (Takara, RR036A, CN). The cDNA was then used as the template to amplify DNA fragments by PCR for the probe synthesis. The PCR products were inserted into the pBlueScript vector by T4 ligation polymerase (Takara, 2040A, CN). The probes were synthesized using the Digoxigenin-labelling Mix (Roche, 11,277,073,910, DE) and T3 RNA polymerase (Roche, 11,031,171,001, DE) or T7 RNA polymerase (Roche, 10,881,175,001, DE). The in situ hybridization was performed as previously described [26, 27].

### Immunofluorescence

Immunofluorescence was performed as previously described [19]. The primary antibodies and dilutions were as follows: anti-Calretinin (Millipore, AB5054, 1:500); anti-Calbindin (Millipore, AB1778, 1:250); anti-Foxg1 (Abcam, ab18259, 1:1000); anti-GFP (Abcam, ab13970, 1:1000); anti-L1 (Millipore, MAB5272, 1:500); anti-Pax6 (BioLegend, 901,301, 1:1000); and anti-Vglut2 (Millipore, MAB5504, 1:500). The secondary antibodies used were Alexa Fluro 488 donkey anti-chicken (Jackson Lab, 703–545-155, 1:500), Alexa Fluor 488 donkey anti-rabbit (Life, A21206, 1:500), Alexa Fluor 546 donkey anti-rabbit (Life, A10040, 1:500), Alexa Fluro 647 donkey anti-rabbit (Life, A31573, 1:500), Alexa Fluor 488 donkey anti-rat (Life, A21208, 1:500), CF 633 donkey anti-rat (Sigma-Aldrich, SAB4600133, 1:500) and Alexa Fluro 647 donkey anti-mouse (Invitrogen, A21236, 1:500).

### Statistical analysis and cell counting

The measurements for the volumetric analyses were performed using every tenth 8 μm coronal section stained with anti-Calretinin. The regions of the habenula were measured using ImageJ software as previously described [28, 29]. The volumes (V) were calculated as V = ∑A × i × d, according to Cavalieri’s principle, where A represents the sum of the areas in the habenula, I represents the intervals between the sections, and d represents the thickness of the sections. The measurements for analysis of the thickness of habenular commissure were performed using every third 10 μm coronal section by the immunofluorescence of L1. The thickness at the midline area were measured by ImageJ software and calculated by the average. Cells of each distinct cell type in the sub-nuclei of the habenula were counted, and the numbers of CR^+^, CB^+^, Tac1^+^ and Pax6^+^ cells were counted in every tenth 8 μm coronal section from each side of the habenula and summed to obtain the total number. We considered every strong Tac1^+^ staining dot as a single cell under high magnification views. Very weak staining was not taken into account. Both controls and mutants were counted under the same criterion. The area of Brn3a^+^ cells in the medial habenula was measured by ImageJ software in every third 10 μm coronal section from one side of the habenula and averaged to obtain the mean area per section. All experiments were performed using at least three different litters, and the data were statistically analysed using GraphPad Prism software. Two-tailed Student’s t-test was performed to analyse the statistical significance at *p* < 0.05 (*), *p* < 0.01(**) and *p* < 0.001(***).

### Quantitative real time polymerase chain reaction (qRT-PCR)

qRT-PCR was carried out according to the protocols as previously described [19]. The dorsal part of E12.5 diencephalon at least from three different litters were used. The specific primers for *Fgf15* is: 5′-GAGGAAGCCAGAAGGTATGAAG-3′ and 5′-GGCAAGCTAAGATCCCATGA- 3′.

## Results

### *Foxg1* is specifically expressed in the developing dorsal diencephalon, and ablation of *Foxg1* leads to an impaired epithalamus

**Fig. 1:**
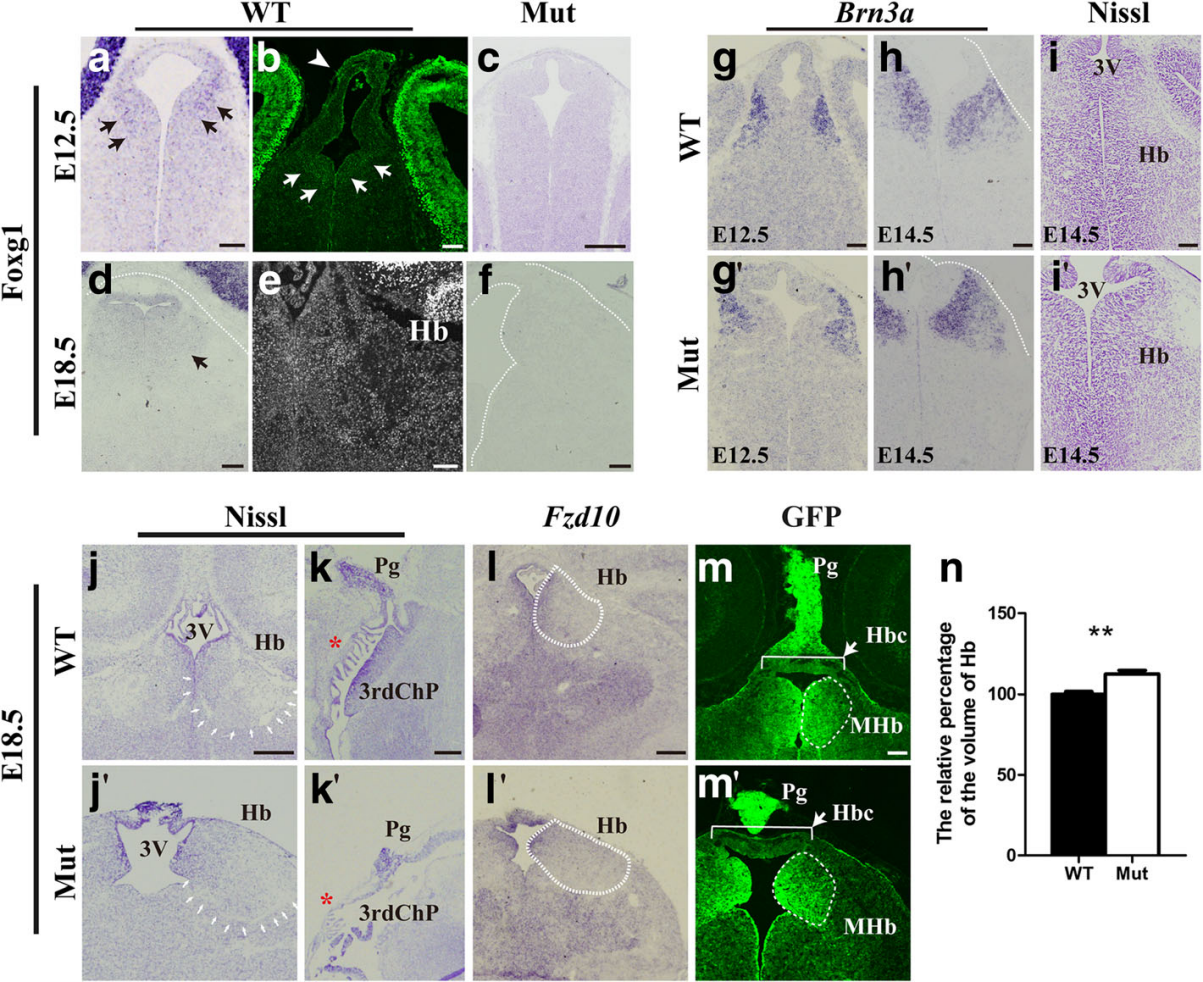
*Foxg1* is required for the development of the epithalamus. (**a** and **b**): In situ hybridization of *Foxg1* (**a**) and immunofluorescence of Foxg1 (**b**) in E12.5 coronal sections. *Foxg1* is expressed at the dorsal developing diencephalic ventricular zone, particularly at the prospective habenular ventricular zone (arrows) and the pineal gland (arrowhead). (**c**): Foxg1 is effectively eliminated in *Foxg1* mutants at E12.5. (**d** and **e**): In situ hybridization of *Foxg1* (**d**) and immunofluorescence of Foxg1 (**e**) in E18.5 coronal sections. Arrow in **d** show the expression of *Foxg1* in the habenula. (**f**): Foxg1 is effectively ablated at E18.5. (**g**-h’): In situ hybridization of E12.5 (**g**-g’) and E14.5 (**h**-h’) forebrain with *Brn3a* showing the habenula were slightly enlarged. (**i**, i’): Nissl staining of E14.5 forebrain in coronal sections revealed the enlarged habenula. The white dashed line in **d**, **f**, **h** and h’ outlined the diencephalon. (**j**-k’): Nissl staining in E18.5 coronal (**j**-j’) and sagittal sections (**k**-k’) showing the dorsolaterally expanded habenula (**j**, j’, arrows), enlarged third ventricle, more branched third ventricle choroid plexus (**k**, k’, asterisk) and the aberrant shape of the pineal gland in mutants. (**l**, l’): In situ hybridization revealed the Fzd10 ^+^ habenular region were enlarged. (**m**, m’): Immunofluorescence with GFP showing the expanded habenula in the mutants (m’) compared to that in the controls (**m**), the elongated habenular commissure (bracket) and the abnormal pineal gland. (**n**): Measurement of the volume of the habenula (*n* = 8, ** *p* = 0.0039). 3^rd^V, third ventricle; 3^rd^Chp, third ventricle choroid plexus; 3^rd^VZ, ventricular zone of the third ventricle; Hb, habenula; Hbc, habenular commissure; MHb, medial habenula; Pg, pineal gland. Scale bars: 100 μm

The forkhead box transcription factor *Foxg1* has been reported to be critical for telencephalic development [17]. However, the function of *Foxg1* in the diencephalon has been under-investigated. Considering the symptoms, including poor sleep patterns, emotional disorders and choroid plexus cysts, observed in patients suffering from FOXG1 syndrome [21–23], we suspect that *Foxg1* plays an important role in the developing diencephalon. Previously, the forced overexpression of *Foxg1* in chicks has been shown to downregulate *Otx2* in the alar plate of the diencephalon, indicating that *Foxg1* likely plays a role in diencephalic development [30]. In this study, we first analysed the expression of *Foxg1* in detail using in situ hybridization. As shown in Fig. 1a, at E12.5, although extremely strong staining was detected in the developing telencephalon, *Foxg1* was also found to be weakly expressed in the progenitors in the third ventricular zone (3^rd^VZ) and their postmitotic derivatives. This expression pattern was confirmed by immunostaining with anti-Foxg1 (Fig. 1b, arrow). Strong staining was particularly detected at the dorsal-most region of the diencephalon, which was presumably the epithalamus area from which the habenula, pineal gland and 3^rd^Chp arise (Fig. 1a, b). As development proceeded, the expression level of *Foxg1* gradually increased with stronger expression in the medial habenula (MHb) and weaker expression in the lateral habenula (LHb) at E18.5 (Fig. 1d, e). Previously, a new *Foxg1-IRES-Cre* line that faithfully recapitulates the endogenous *Foxg1* expression also exhibited Cre-medicated recombination in the developing epithalamus [31], which is consistent with our observations. Collectively, *Foxg1* is specifically expressed in the developing diencephalon, particularly in the dorsal part of the 3^rd^VZ, strongly supporting that *Foxg1* plays a role in the development of the epithalamus.

Subsequently, we adopted the “self-deletion” approach by crossing *Foxg1-Cre* with *Foxg1*^*fl/fl*^ to obtain compound homozygous *Foxg1*^*cre/fl*^ mice [32]. Both *Foxg1*^*cre/fl*^ and *Foxg1*^*cre/cre*^ were considered *Foxg1*-phenotypical null mutants and in this study, all results were obtained in comparable levels from serial sections of the habenula. As shown in Fig. 1c and f, the expression of *Foxg1* in the dorsal diencephalon were effectively eliminated in the mutants at E12.5 and E18.5. We first analysed the changes of the epithalamus during early development. Previously, *Brn3a* (also called *Pou4f1*) has been reported to be strongly expressed in postmitotic neurons in the MHb and weakly expressed in the LHb and critical for the development of the habenula [33]. At the stages of E12.5 and E14.5, in situ staining of *Brn3a* showed the habenula was slightly enlarged and expanded dorsal-laterally after *Foxg1* deletion (Fig. 1g, g’; h, h’). This is confirmed by Nissl staining as well (Fig. 1i, i’). At E18.5, the habenula visualized by Nissl staining expanded much more than that of observed in E12.5 and E14.5. The third ventricle was found to be significantly enlarged which could be a result of the changes caused in the lateral ventricle due to the decrease in the size of the cortex as previously reported [34, 35] (Fig. 1j, j’). However, the pineal gland was smaller (Fig. 1k, k’). Interestingly, the 3^rd^Chp were more branched in *Foxg1* mutants compared to the controls (Fig. 1k, k’ , asterisk). To further confirm these abnormalities, we performed in situ hybridization for *Frizzled10* (*Fzd10*), one of the Wnt receptors, specifically expressed in the MHb progenitors and postmitotic neurons [36]. As shown in Fig. 1l-l’ , Fzd10^+^ region was enlarged. We also generated a *Fzd10-EGFP* transgenic mouse line in which EGFP faithfully reflected the endogenous Fzd10 expression (unpublished data). As shown in Fig. 1m-m’ , the MHb appeared to expand to the lateral side, resulting in an irregular shape, and the habenular commissure was lengthened and became thinner. A smaller pineal gland was also observed, which is consistent with the observations using Nissl staining. Finally, we evaluated the volume of the habenula and found that it was relative increased by approximately 12% in mutants (Fig. 1n). Collectively, the disruption of *Foxg1* caused an impaired epithalamus.

### Increased numbers of epithalamic progenitors and habenular subtype neurons

**Fig. 2:**
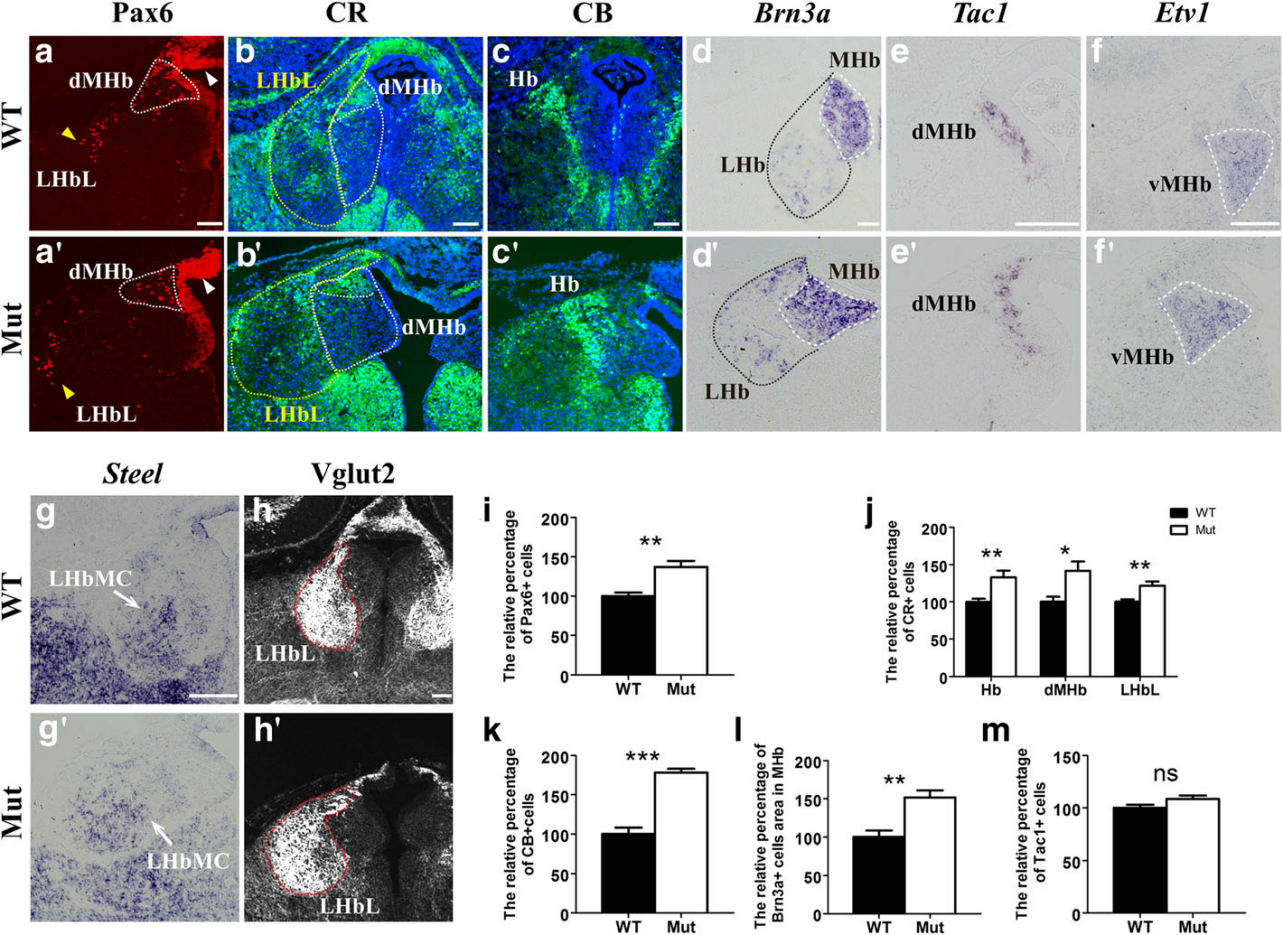
Abnormal development of the habenular subtype neurons. (**a**-a’): Immunofluorescence of anti-Pax6. Pax6^+^ progenitors in the epithalamic VZ (white arrowhead) were remarkably increased compared to those in the controls, and more Pax6^+^ cells were scattered in the dMHb (dotted line) and LHbL (yellow arrowhead). (**b**-b’): Increased number of CR^+^ subtype neurons in the dMHb (white dotted line) and LHbL (yellow dotted line). (**c**, c’): Increased numbers of CB^+^ subtype neurons in the habenula. (**d**, d’): In situ hybridization showing *Brn3a* strongly expressed in postmitotic neurons in the MHb (white dotted line) and weakly expressed in the LHb (black dotted line), and both stained areas were expanded with irregular morphologies in the *Foxg1* mutants. (**e**-f’): Substance P-ergic neurons in the dMHb showing *Tac1* in situ hybridization and Etv1^+^ glutamatergic neurons in the vMHb. (**g**-h’): Enlarged LHbMC and LHbL. (**i**-**m**): Quantitative analysis of the numbers of Pax6^+^ progenitors (**i**, *n* = 6, **p = 0.002), CR^+^ neurons (**j**, n = 6, Hb, **p = 0.0085; dMHb, *p = 0.0148; LHbL, **p = 0.0069), CB^+^ neurons (**k**, n = 6, ***p < 0.0001), and Tac1^+^ neurons (**m**, n = 8, *p* = 0.067). (**l**) Quantitative analysis of Brn3a^+^ area in coronal section crossing the midmost level of the habenula (**l**, *n* = 4, **p = 0.0063). The data are presented as the mean ± S.E.M. Hb, habenula; MHb, medial habenula; dMHb, dorsal medial habenula; vMHb, ventral medial habenula; LHb, lateral habenula; LHbL, lateral division of the lateral habenula; LHbMC, central part of the medial division of the lateral habenula. Scale bars: 100 μm

To further investigate the cause of the enlarged habenula following the *Foxg1* deletion, we examined the progenitor pool at E18.5. In the controls, Pax6 was expressed in the dorsal 3^rd^VZ, and its expression was particularly intense in the epithalamic progenitors. A portion of the Pax6^+^ progenitor cells was also dispersed within the dorsal MHb (dMHb) (Fig. 2a, dotted line) and the lateral division of the LHb (LHbL) (Fig. 2a, yellow arrowhead). In the mutants, the epithalamic VZ seemed thicker than that in the controls, and more Pax6^+^ cells were scattered in the dMHb and LHbL; the total number of Pax6^+^ cells was significantly increased by approximately 37% (Fig. 2a, a’ , i), demonstrating that disruption of *Foxg1* results in an increased number of progenitors in the developing epithalamus. Then, we examined the alterations in several subtypes of habenular neurons. *Calretinin* (*CR*) and *Calbindin* (*CB*) are two members of the EF-hand family of calcium-binding proteins that are required for the differentiation of early generated thalamic neurons [37]. Here, we found that CR^+^ neurons were mainly populated in the dMHb and LHbL, which are the similar regions in which the Pax6^+^ progenitors were located; additionally, the CR^+^ neurons were distributed in the central part of the medial division of the LHb (LHbMC). The total number of CR^+^ cells in the habenula was remarkably increased by approximately 33%, a 42% increase in the dMHb and a 22% increase in the LHbL were observed (Fig. 2b, b’; j). The CB^+^ neurons were mainly detected in the boundary between the MHb and the LHb. In the mutants, the number of CB^+^ neurons was also significantly increased by approximately 78%, and more CB^+^ neurons were scattered in the LHb (Fig. 2c, c’; k). In situ staining showed Brn3a^+^ areas in both the mutant MHb and LHb were expanded (Fig. 2d, d’). We measured the Brn3a^+^ area at the central level of the MHb and found there was approximate 51% increase in mutants (Fig. 2l).

We further analysed the changes in several other neuronal subtypes in the MHb and LHb. The dMHb has been previously shown to contain a group of neurons that release the neuropeptide substance P, and the ventral part of the MHb (vMHb) contains glutamatergic neurons [5, 38]. According to the in situ staining of *Tachykinin1* (*Tac1*), which acts as a precursor of substance P, the number of substance P-ergic neurons was comparable to that in the controls (Fig. 2e, e’; m). However, *Etv1*, which is a member of the ETS family of transcription factors, was specifically expressed in a partition of the habenular glutamatergic neurons [33], which was significantly enlarged (Fig. 2f, f’). Regarding the LHb subdivisions, we examined the LHbMC and LHbL. As visualized by the staining of *Steel*, the ligand for the receptor tyrosine kinase c-kit and type 2 vesicular glutamate transporter (*Vglut2*), the mutant Steel^+^ LHbMC and Vglut2^+^ LHbL expanded more broadly than the controls. However, the expression level of Steel seemed less compared with the controls (Fig. 2g-h’). Collectively, the disruption of *Foxg1* led to an increased number of progenitors in the developing epithalamic VZ, which may ultimately result in defects in subtype neurons in both the MHb and LHb.

### Impaired habenular innervations after *Foxg1* deletion

**Fig. 3:**
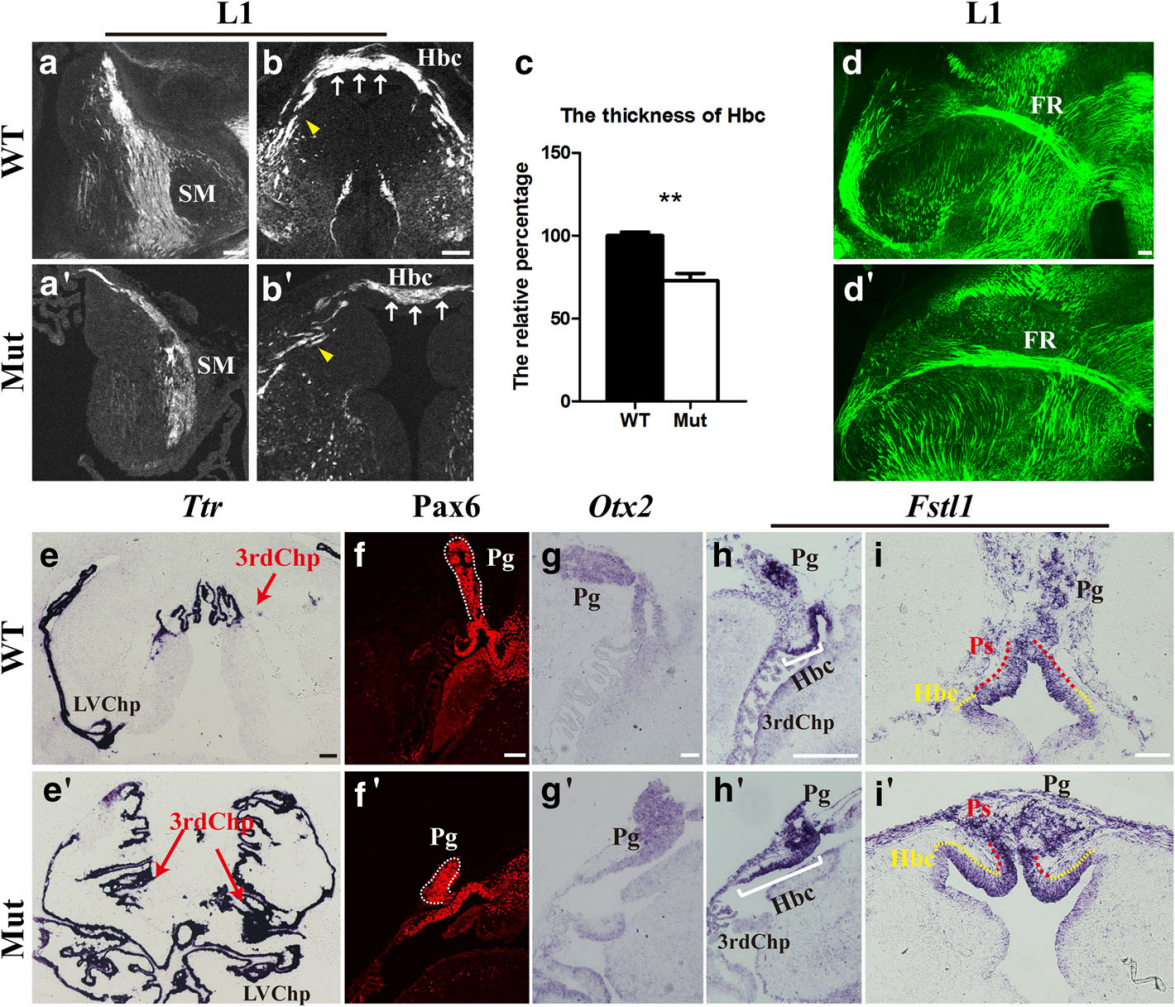
Disrupted habenular innervations and abnormal diencephalic Chp and pineal gland. (**a**-b’): Immunofluorescence of L1 showing a reduced SM and habenular commissure (arrows in **b** and b’). Arrowhead indicates poorly fasciculated projections in the mutants compared with those in the controls. (**c**): Quantitative analysis of thickness of the habenular commissure at the midline area (*n* = 4, **p = 0.0015). (**d**, **d**’): less fasciculated FR and slightly changed projection angle. (**e**, e’): In situ hybridization of *Ttr* showing a more branched choroid plexus in the mutants. (**f**-i’): Immunofluorescence with Pax6 (**f**, f’) and in situ hybridisation of *Otx2* (**g**, g’) and *Fstl1* (**h**-**i**’) revealing an aberrant pineal gland, a shortened pineal stalk, which links the pineal gland to the habenula (**i**, i’, red broken line), and a lengthened Hbc area (**h**, h’, bracket; **i**, i’, yellow broken line). SM, stria medullaris; 3^rd^Chp, third ventricle choroid plexus; LVChp, lateral ventricle choroid plexus; FR, fasciculus retroflexus; Hb, habenula; Hbc, habenular commissure; IPN, interpeduncular nucleus; Pg, pineal gland; Ps, pineal stalk. Scale bars: 100 μm (scale bar in **g** and g’: 400 μm)

Due to the remarkable structural alteration in the mutant epithalamus, we investigated the changes in the innervations. Immunostaining of anti-L1, which is a neural cell adhesion molecule, was performed. As shown in Fig. 3a-a’ , the stria medullaris (SM), which project forebrain inputs to the habenula [39], were dramatically impaired, which may also be a consequence of the severely impaired telencephalon in the mutants. The habenular commissure, which conveys information between the paired habenulas, was much thinner than that in the controls, although it could cross the midline (Fig. 3b, b’). The control processes were well fasciculated and projected dorsally across the midline. However, the mutant processes were poorly fasciculated and significantly decreased. The decrease was consistent throughout the rostro-caudal axis when observed in serial sections. We then analysed the thickness of habenular commissure in the midline area and found it was reduced by approximate 28% in *Foxg1* mutants (Fig. 3c). The dramatically decreased habenular commissure was also detected by immunostaining with anti-CR and Vglut2 at E18.5 as shown in Fig. 2b, b’; h, and h’. Despite the increased numbers of neuron subtypes, the severely decreased habenular commissure indicates that neuronal differentiation may be affected by the *Foxg1* deletion as well.

Interestingly, the fasciculus retroflexus (FR), through which the habenula projects to the interpeduncular nucleus (IPN) of the midbrain [39, 40], appeared to project correctly, although it was less fasciculated and its projecting angle was slightly changed, which may be due to the irregular morphology of the habenula (Fig. 3d, d’). Thus, *Foxg1* is essential for the development of habenular innervations, particularly the habenular commissure.

### Reduced pineal gland and extremely complicated Chp after *Foxg1* deletion

During development, both the pineal gland and the 3^rd^Chp, along with the habenula, arise from the dorsal region of p2, which is the presumptive epithalamic domain. In addition to the habenula, the pineal gland and 3^rd^Chp were also impaired in *Foxg1* null mutants. According to the in situ hybridization of *Ttr*, the 3^rd^Chp and lateral ventricle Chp displayed extremely complicated morphologies compared to those in the controls (Fig. 3e, e’) and, to a certain extent, reflected the Chp cyst in FOXG1 patients. Meanwhile, the size of the pineal gland was reduced viewed by immunostaining of Pax6 (Fig. 3f, f’). Previous studies have shown that misregulation of *Foxg1* in chick prosencephalon causes the downregulation of *Otx2,* which is required for the development of the pineal gland and Chp [30, 41, 42]. However, here, we found that in the absence of *Foxg1*, the expression level of *Otx2* in the pineal gland appears normal (Fig. 3g, g’), indicating that *Otx2* is not involved in the *Foxg1*-regulated development of the pineal gland in mice. We observed strong expression of Follistatin-like 1 (Fstl1), which is a secreted glycoprotein that functions as an antagonist of BMP signalling in the developing pineal gland [43, 44]. As shown in Fig. 3h-i’ , at E18.5 in the controls, *Fstl1* was densely expressed in a distinct cell population in the pineal gland, pineal stalk, and habenula commissure. However, in the mutants, the Fstl1^+^cells were not well organized, and the Fstl1^+^ pineal stalk was remarkably shortened with a lengthened habenula commissure. In summary, *Foxg1* may be essential for the regional identities of the dorsal part of p2.

### Early sub-regionalization of the presumptive epithalamic domain was disrupted after *Foxg1* deletion

**Fig. 4:**
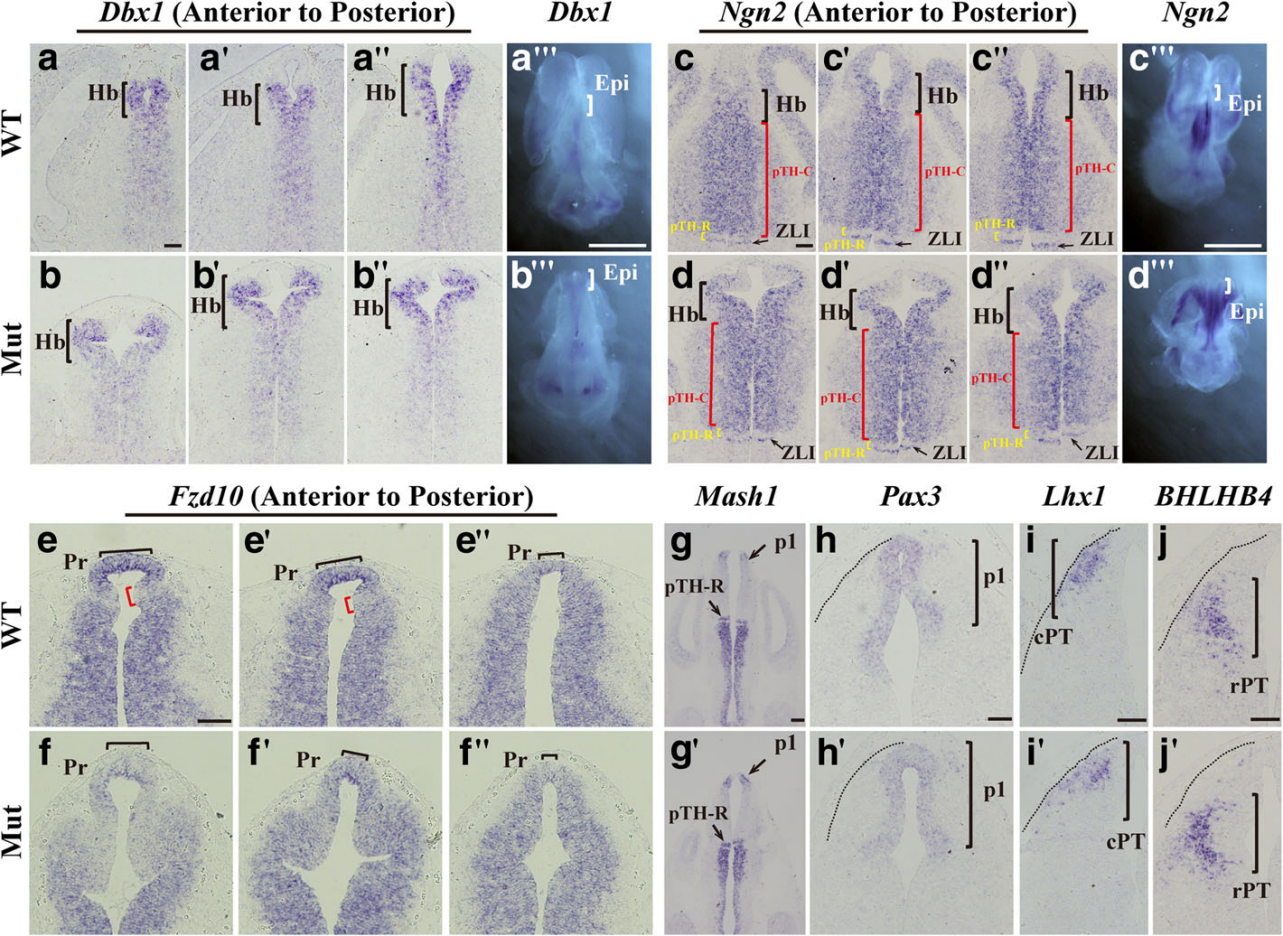
*Foxg1* is required for early epithalamic sub-regionalization. (**a**-**b**”): In situ hybridization of *Dbx1* showing that the habenular progenitor region (bracket) was obviously expanded and shifted dorsolaterally after *Foxg1* deletion. (**a**”’, **b**”’) Whole-mount in situ hybridization for *Dbx1* at E12.5 The bracket indicates the epithalamus. (**c**-**d**”): Expanded Ngn2^+^ habenular VZ (black bracket) but normal pTH-C (red bracket), pTH-R (yellow bracket) and ZLI (arrow) in the mutants. (**c**”’, **d**”’): Whole-mount in situ hybridization for *Ngn2* at E12.5. The bracket indicates the epithalamus. (**e**-**f**”): Smaller pineal recess (black bracket) shown by in situ staining of *Fzd10*. The red bracket marked the *Fzd10*^*weak*^ strip between the pineal recess and the habenular ventricle zone. (**g**-g’): In situ hybridization of *Mash1* showing normal pTH-R (arrow) and ZLI. (**h**-**j**’): No obvious changes in the patterning of p1 were revealed by the in situ staining with *Pax3*, which labels the pretectal VZ (**h**, h’); *Lhx1*, which labels the mantle zone of the caudal pretectum (**i**, i’); and *BHLHB4*, which labels the mantle zone of the rostral pretectum (**j**, **j**’). The black dashed line in **h**-**j**’ outlined the diencephalon. Epi, epithalamus; Hb, habenula; pTH-C, caudal progenitor domain of the thalamus; pTH-R, rostral progenitor domain of the thalamus; Pr, pineal recess; p1, prosomere 1; cPT, caudal pretectum; rPT, rostral pretectum; ZLI, zona limitans intrathalamica. Scale bars: 100 μm (scale bar in **a**”’, **b**”’, **c**”’ and **d**”’: 2 mm; **g** and g’: 200 μm)

To further examine whether the regionalization of the epithalamic domain was affected by the loss of *Foxg1*, in situ hybridization was performed during the early developmental stage at E12.5. As shown in Fig. 4a-a”, the high-level expression of the homeodomain gene *Dbx1* normally demarcates the habenular progenitor region [45]. In the absence of *Foxg1*, the Dbx1^+^ domain was obviously expanded and shifted dorsolaterally (Fig. 4b-b”). The whole-mount in situ hybridization further confirmed the expansion of *Dbx1* in *Foxg1* mutants (Fig. 4a”’ and b”’). *Ngn2*, which is a member of the proneural bHLH transcription factor family, has been shown to be widely expressed in the habenular VZ, caudal progenitor domain of the thalamus (pTH-C), and key diencephalic organizer zona limitans intrathalamica (ZLI) which is located at the interval between p2 and p3, but specifically excluded from the rostral progenitor domain of the thalamus (pTH-R) in controls [46] (Fig. 4c-c”). In the mutants, the Ngn2^+^ habenular VZ was expanded, while ZLI, pTH-C and pTH-R appeared comparable to those in the controls (Fig. 4d-d”). The similar results were obtained by the whole-mount hybridization (Fig. 4c”’ and d”’). Next, we examined whether the primordium of the pineal gland was affected by the in situ staining of *Fzd10* and found that the mutant pineal recess was obviously smaller than that in the control (Fig. 4e-f”). Thus, sub-regionalization in the developing epithalamus was severely impaired.

To further investigate whether *Foxg1* affects pTH-C, pTH-R and ZLI, in situ hybridization of *Mash1* was also performed. As shown in Fig. 4g-g’ , *Mash1* was strongly expressed in pTH-R and p3 but specifically excluded from the ZLI [46]. There were no differences to be detected between the control and the mutant, consistent with that viewed by in situ hybridization of *Ngn2* (Fig. 4c-d”). We further analysed the patterning of p1 by *Pax3*, which labels the pretectal VZ [47]; LIM-homeodomain transcription factor 1 (*Lhx1*), which is a commonly used marker of the mantle zone of the caudal pretectum [48]. *BHLHB4*, which is a member of the basic helix-loop-helix (bHLH) family; and a specific marker for the mantle zone of the rostral pretectum [49]. No obvious alterations were observed (Fig. 4h-j’). Collectively, *Foxg1* is required for the sub-regionalization of the presumptive epithalamic domain but has no effects on the other parts of p2 and p1 during early diencephalic development.

**Fig. 5:**
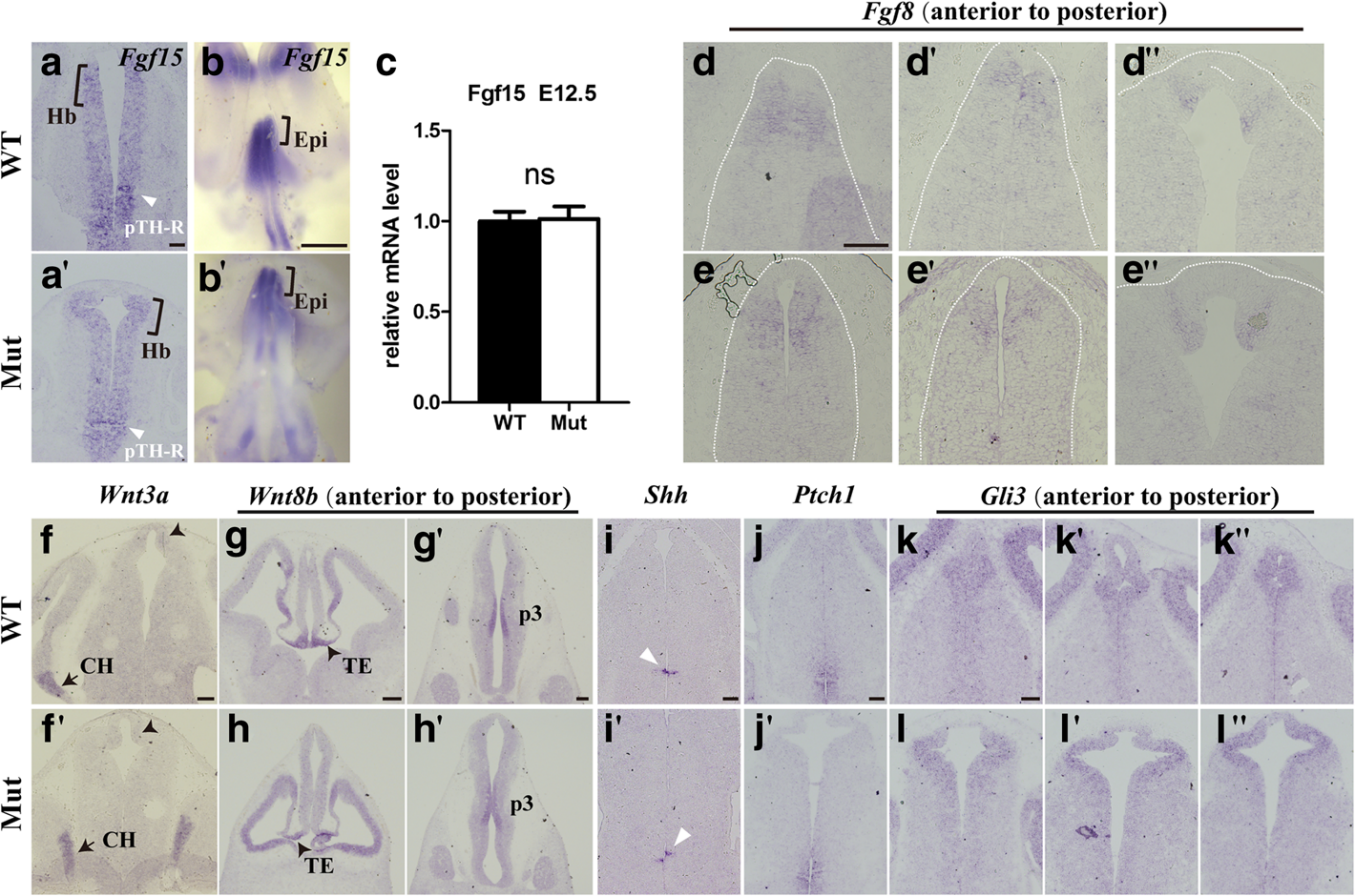
Shh and Fgf signalling were not affected in the epithalamic development. (**a**-a’): Transcription level of *Fgf15* in the presumptive habenula (bracket) was not obviously affected in the mutants, no changes were observed in the region of pTH-R (arrowhead) in p2. (**b**-b’): The whole mount in situ from E12.5 embryos also showing a comparable transcription level in the epithalamus (bracket). **c**: Relative mRNA levels of *Fgf15* (*n* = 4, *p* = 0.898). (**d**-**e**”): No obvious differences in *Fgf8* were observed in the *Foxg1* mutants. The white dashed line outlined the diencephalon. (**f**-**h**’): Staining of *Wnt3a* in dorsal P2 and *Wnt8b* in TE and P3 in mutants were comparable to that of controls. Arrows in **f** and f’ indicate the cortical hem, arrowheads in **g** and **h** indicate the thalamic eminence. (**i**-**l**”): The activity of the Shh signalling pathway appeared normal in the mutants. The white arrow in **i** and **i**’ indicates the ZLI. CH, cortical hem; Epi, epithalamus; Hb, habenula; IPN, interpeduncular nucleus; TE, thalamic eminence; ZLI, zona limitans intrathalamica. Scale bars: 100 μm (scale bar in **b** and b’: 1 mm, **g**-**h**’: 200 μm)

### Shh and Fgf signalling were not affected during the development of the epithalamus

Multiple signals, including fibroblast growth factor (Fgfs) are expressed in the dorsal midline of the diencephalon [15] and coordinate with Shh, which is secreted from the ZLI and basal plate, to establish regional identity in the developing diencephalon [50, 51]. *Fgf8* has been reported to be involved in the patterning of the p2 region. In *Fgf8* hypomorphic mice, the pineal gland and habenula are lost or reduced, exhibiting dose-dependent changes [15]. *Fgf15*, which is another member of the Fgf family, has been shown to act as a downstream target of *Shh* that regulates the development of the diencephalon [52]. Using in situ hybridization, we explored whether Fgf signalling is involved in the regulation of *Foxg1* in the sub-regionalization of the epithalamic domain. As shown in Fig. 5a, at E12.5, *Fgf15* was expressed in the future habenula and thalamic VZ with no detectable expression in the developing pineal gland and 3^rd^Chp in the controls; the transcription level of *Fgf15* in the mutant presumptive habenula was comparable to that of controls, no significant changes were observed in p2 (Fig. 5a, a’ , arrowhead). The same result was obtained using whole-mount hybridization and qRT-PCR (Fig. 5b, b’; c). No detectable changes were observed in *Fgf8* either along the AP axis (Fig. 5d-d”; e-e”). Therefore, *Fgf15,* as well as *Fgf8,* were not involved in the *Foxg1*-mediated regulation of the development of the epithalamus. Previously *Wnt3a* has been reported to be expressed in the dorsal p2 and *Wnt8b* is expressed in the prethalamus [15]. *Foxg1* has been shown to suppress Wnt function in the developing telencephalon [15, 53]. To explore whether *Foxg1* regulate the development of epithaluams through Wnt signalling, we then examined *Wnt3a* and *Wnt8b*; however, no obvious alteration were detected either (Fig. 5f-h’).

Shh, which is secreted by the ZLI, is critical for the development of the diencephalon [50, 54–56]. The ventral ^high^-dorsal^low^ gradient of Shh specifies the diencephalic regional identity [57]. Therefore, we examined whether Shh signalling contributes to the epithalamic defects. At E12.5, in the *Foxg1* mutants, the expression of *Shh* in the ZLI was comparable to that in the controls (Fig. 5i, i’). We then examined the activity of the Shh signalling pathway as reflected by *Ptch1* [58]. No obvious differences were detected (Fig. 5j, j’). Meanwhile, the level of *Gli3*, which is a member of the Glioma-associated oncogene (*Gli*) family that has been reported to inhibit Shh signalling [59], also appeared normal in the epithalamic VZ (Fig. 5k-k”; l-l”). Collectively, *Foxg1* may regulate the development of the epithalamus independently of Shh signalling.

## Discussion

*Foxg1* has been reported to be critical for telencephalic development [17, 19]. However, its role in the development of the diencephalon remains unclear. Individuals with FOXG1 syndrome exhibit a disturbed sleep pattern, Chp cysts and emotional disorders [21–23], suggesting that *Foxg1* likely plays a role in epithalamic development. In this study, we demonstrate that *Foxg1* is essential for the development of the epithalamus. The disruption of *Foxg1* leads to an extremely complicated Chp, a reduced pineal gland and an enlarged habenula. Moreover, we demonstrate that *Foxg1* may be required for the regional specification of the epithalamic progenitor domain independently of Shh and Fgf signalling. Our findings shed light on the molecular mechanism underlying the subdivision of the epithalamic domain.

Previously, the function of *Foxg1* was under-investigated in the developing diencephalon. In this study, we identified specific expression of *Foxg1* in the dorsal part of p2 from which the epithalamus derives and further elucidated its function during epithalamic development. The habenula has been reported to be closely related to emotional disorders and has recently attracted increasing attention [2, 9, 10]. By receiving inputs from the limbic system and basal ganglia and projecting to monoaminergic nuclei, the habenula acts as a node that connects the forebrain to the brainstem [39]. Here, we found that the disruption of *Foxg1* results in an enlarged habenula with an increased number of subtype habenular neurons. The loss of *Foxg1* also caused differentiation defects in habenular neurons, which led to impaired habenular innervations and ultimately resulted in abnormal information conveyance among the forebrain, brainstem and paired habenula, which may account for the emotional disorders observed in patients with *FOXG1* mutations. To the best of our knowledge, this is the first report illustrating that *Foxg1* regulates the development of the epithalamus.

During development, the most dorsal domain of p2 gives rise to the epithalamus, which consists of 3^rd^Chp, pineal gland, habenular commissure and habenula. However, the mechanism by which the sub-regional identities are established is unknown. Multiple signals, including Fgfs have been found to be specifically expressed in the dorsal region of p2 and involved in the development of the epithalamus [15, 60, 61]. Previous studies have shown that the pineal gland and habenula are lost or reduced in *Fgf8* hypomorphic mice, which exhibit dosage-dependent changes in the epithalamus [15]. Previously, we have reported a strong expression of *Fstl1* in the pineal gland [43]. In this study, we have detected *Fstl1* is also expressed in the habenular commissure. The remarkably shorten pineal stalk with the lengthened habenular commissure observed in the *Foxg1* mutants indicate *Fstl1* may be required for the development of the pineal gland and the habenular commissure. Further study is needed to elucidate its function. Shh is secreted from the ZLI and basal plate, and by coordinating with signals from the dorsal region, *Shh* is critical for the regionalization of the diencephalon [45, 50, 51]. *Fgf15* has been identified as a downstream target of *Shh* that suppresses cell proliferation and promotes differentiation in the developing telencephalon [62]. In this study, we did not detect obvious changes in Shh and Fgf signalling in our mutants. The mutant ZLI was comparable to that in the controls, and the activity of the Shh signalling pathway, as shown by *Ptch1* and *Gli3,* appeared normal, suggesting that the regulation of *Foxg1* during epithalamic development is independent of Shh and Fgf signallings. Further studies are required to determine the downstream targets of *Foxg1* during the development of the epithalamus.

## Conclusions

In the present study, we have identified a specific expression of *Foxg1* in the developing epithalamus and further found that disruption of *Foxg1* resulted in an impaired epithalamus with an expanded habenula, a reduced pineal gland and more branched choroid plexus. No obvious changes in Shh and Fgf signaling were detected in *Foxg1* mutants, indicating that *Foxg1* may regulates the development of the epithalamus independent of Shh and Fgfs. Our findings provide new insights into the regulation of the development of the epithalamus. Further study is required to elucidate the molecular mechanism.

## Abbreviations

3rdChp: Third ventricle choroid plexus
3^rd^V: Third ventricle
3^rd^VZ: Ventricular zone of the third ventricle
CH: Cortical hem
cPT: Caudal pretectum
dMHb: Dorsal medial habenula
FR: Fasciculus retroflexus
Hb: Habenula
Hbc: Habenular commissure
IPN: Interpeduncular nucleus
LHb: Lateral habenula
LHbL: Lateral division of the lateral habenula
LHbMC: Central part of the medial division of the lateral habenula
MHb: Medial habenula
p: Prosomere
Pg: Pineal gland
Pr: Pineal recess
Ps: Pineal stalk
pTH-C: Caudal progenitor domain of the thalamus
pTH-R: Rostral progenitor domain of the thalamus
rPT: Rostral pretectum
SM: Stria medullaris
TE: Thalamic eminence
vMHb: Ventral medial habenula
ZLI: Zona limitans intrathalamica
